# Shear dominated deformation with curved beaks in folding–shearing

**DOI:** 10.1007/s00170-025-15853-9

**Published:** 2025-06-11

**Authors:** Rishabh Arora, Omer Music, Julian M. Allwood

**Affiliations:** 1 Department of Engineering, University of Cambridge, Trumpington Street, Cambridge, CB2 1PZ UK; 2 DeepForm Ltd., Allia Future Business Centre, Cambridge, CB4 2HY UK

**Keywords:** Sheet metal forming, Folding-Shearing, Stamping, Design optimization, Tool geometry

## Abstract

The deep drawing process in the automotive industry generates up to 45% material waste. To address this issue, the folding–shearing process was developed as a drop-in solution, enabling the formation of parts in pure shear with minimal thickness variation. This process involves folding a blank while collecting the excess material in a region called the ‘beak’, which is subsequently sheared in-plane using a single set of tools moving in one forming direction. This paper investigates the extent to which the curvature of the geometry of the beak influences the resulting thickness distribution. A combination of physical and numerical trials demonstrates that a beak design with a negative Gaussian curvature reduces the maximum thickening by 65%. This reduction in thickening helps minimise the forming loads and tool wear, thereby improving the overall robustness of the process. An analytical model is proposed to predict the resulting thickness distribution and demonstrates accuracy within a 12.5% deviation from experimental results. Finally, a design map is proposed to instantly identify the optimal beak design parameters without the need for extensive numerical or physical validations while ensuring a minimal thickness change.

## Introduction

As much as 12% of steel and 30% of aluminium produced globally are used in the automotive industry [1]. These materials contribute to approximately one-third of the vehicles’ embodied emissions [2], a significant concern given the ongoing climate crisis. Researchers are driven to address these emissions by pursuing lightweight designs [3], optimising process parameters [4], advancing manufacturing technologies such as near-net casting [5], minimising material usage by blank shape optimisation [6], or by using tailor welded blanks [7]. In the automotive industry, the majority of current sheet metal forming is done by deep drawing, where a flat metal blank is clamped along its perimeter in the blankholder region, while a male punch deforms the metal blank to form the target geometry [8]. Despite its widespread adoption, the process is wasteful, and 45% of the sheet metal purchased by the automotive industry becomes manufacturing scrap [2]. This arises primarily from the use of extra material gripped in the blankholder region to control thinning and thickening limits. Research has shown that this scrap can be reduced and that the production efficiency can be maintained while controlling springback [9], surface quality, and tool wear [10]. However, addressing these challenges requires tailored solutions, which are difficult to investigate in an industrial setting due to strict delivery timelines and complex part geometries. Much research to date has therefore focused on improving the recycling potential of automotive scrap [11], but a more effective approach is to prevent the waste in the first place, yielding both financial and environmental advantages [12].

**Fig. 1:**
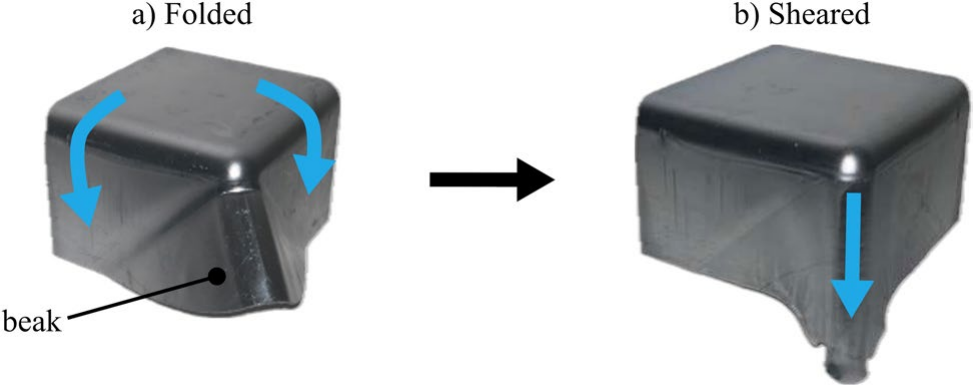
Concept of folding-shearing

To improve material utilization, Allwood et al. [13] invented the folding-shearing method in 2019 and developed an experimental rig to validate the shearing stage, as a pre-cursor to demonstrating the complete process. This process, unlike conventional spinning, can form asymmetric parts at a high production volumes and speeds. Furthermore, in contrast to deep drawing, folding-shearing enables near-net shape forming with minimal scrap because it does not require a blankholder. Cleaver et al. [14] subsequently investigated the complete folding-shearing process as a two-stage method to form a quarter-square cup as shown in Fig. 1.

The process involves two stages: folding and shearing. Firstly, a blank is folded over a radius, while excess material is gathered in regions of incompatibility to form an intermediate shape called the ‘beak’ (Fig. 1(a)). Secondly, the material in the beak is deformed through an in-plane shearing process to form the target geometry (Fig. 1(b)). In addition to saving scrap, Cleaver et al. [14] demonstrated that, for a square cup, folding-shearing achieves 2.5-fold deeper parts compared to deep drawing. Arora et al. [15] later demonstrated forming half a U-channel using a single toolset in one direction and defined a process operating window between the limits of springback, thinning, and thickening. Successful operation requires balancing between excessive thinning, which can lead to tearing, and excessive thickening, which can cause the workpiece to seize between the tools. Therefore, thickness control is critical in determining process success. This paper aims to understand the extent to which the folding-shearing process can be adjusted to control the thickness distribution.

## Literature review

There are significant benefits to thickness-neutral forming as it can in theory occur in a state of pure shear stress, which lowers the forming forces by minimizing the hydrostatic pressures on the workpiece [16]. This in turn decreases the tool wear, thereby increasing the tool longevity and reducing costs. Although thickness variation is inevitable in sheet metal forming, some processes achieve better control through process design and tool geometry. The aim of this literature review is to explore the strategies for better thickness control, especially the influence of curvature at the deformation zone. The review draws inspiration from three conventional processes: conventional spinning, incremental sheet forming, and deep drawing.

Conventional metal spinning forms hollow, axially symmetric geometries. A workpiece is clamped against a conical mandrel on a spinning lathe, while a roller deforms the blanks either in a single step or incrementally [17]. A key characteristic of conventional spinning is that the roller deformation zone exhibits a saddle-like profile, leading to a local negative Gaussian curvature. This curvature results in balanced tensile radial and compressive circumferential stresses, promoting a near-pure shear state that helps maintain a consistent thickness [18]. The common defects in spinning are wrinkling and radial or circumferential cracks [19], and several control features help to mitigate these problems. Existing strategies have considered how a multi-pass process reduces wrinkling [20], while others have explored how the toolpath design [21] and changes in roller geometry [22] can minimise edge cracks. Another approach has investigated how the feed ratio balances the thinning and wrinkling defects, as low feed ratios tend to induce excessive thinning whereas high feed ratios increase the likelihood of wrinkling [23]. However, most existing optimization strategies have focussed on controlling failure rather than leveraging the Gaussian curvature as a thickness-control mechanism. Achieving deformation in pure shear requires the in-plane strains to be equal and opposite in the deformation zone [18], suggesting that the fundamental principle of local curvature may play a direct role in thickness control. However, this relationship has not been extensively explored in metal forming literature.

The impact of Gaussian curvature is evident in other sheet forming processes. For instance, in incremental sheet forming, the deformation zone typically exhibits a positive Gaussian curvature, which induces tensile strains in both in-plane orthogonal directions, leading to thinning [24]. Incremental sheet forming operates by using a rigid tool that follows a spiral toolpath of increasing depths, controlled via a computer numerical control (CNC) machine, gradually accumulating highly localised plastic strains to form complex geometries [25]. The most widely accepted tool is a hemispherical indenter [25], which generates these tensile strains. To mitigate this, researchers have explored alternative incremental sheet forming strategies. For instance, a water-jet incremental sheet forming system was found to reduce friction at the interface [26], while an incremental sheet hammering set-up improved the surface quality [27]. Additional thickness control strategies focus on modifying the toolpath design. For example, controlling the step-down of the tool increases formability [28] while higher rotational speeds enhance surface quality [29]. However, these techniques primarily address secondary effects rather than directly controlling the deformation mechanics dictated by Gaussian curvature.

While thickness changes in incremental sheet forming and spinning arise from curvature-induced strain states, deep drawing, the process which folding-shearing aims to replace, controls thickness by regulating the material flow into the die. This occurs via a series of control mechanisms including blankholder force, draw bead geometry, blank shape, and die design. The *blankholder* applies a force to grip the material such that sufficient tension is provided to control the process window between wrinkling and tearing [8]. On average, this force is approximately 0.5–1.0% of the drawing force [30]. Instead of applying a constant force, varying the blankholder force throughout the stroke [31], implementing spatially adaptive controls [32], and pulsating blankholder force strategies [33] have enhanced thickness control. However, the blankholder force control strategy is typically adjusted reactively at the onset of thinning failure rather than being proactively designed to minimise the thickness variation from the outset [30]. Next, *draw beads* are physical features in the blankholder region that increase the force restraining sheet draw-in [8]. Studies investigating the role of draw bead design and placement have shown their effectiveness for deep parts [34] and complex geometries [35], while they are less critical for shallow or simple components [36]. In industry, the draw bead design is chosen from a dataset, which is later modified during the tool prototype stage and is often developed as a trial-and-error process [37]. Similarly, the *blank* size and shape are an iterative process, and research focuses on minimising the blank size to reduce material usage [38]. Contours of the blank shape are defined, and extra material is added in regions that are more likely to thin, while lesser material is added in the region where the workpiece is more likely to wrinkle [39]. These contours are well researched; however, in industry, blank design also incorporates holes and slits to improve material flow, although this has not been documented in detail in the literature. More recently, the concept of tailor welded blanks has been investigated where, prior to folding, two or more base sheet metals are welded in one plane [40]. These blanks improve product quality by distributing thickness so that more formable material is placed strategically in regions of high stretching and, conversely, less material is used where less deformation is needed. Research continues focussed on welding [41] and assessing the formability limits [42], which are both essential for industrial applicability. Finally, *die design* is well documented for simple geometries such as square or cylindrical cups and U-channel parts. The punch and die radii are found to have an impact on final thickness [8]. The punch radius is a function of the product parameters, and the die radius is a process parameter. It is agreed that a small die radius results in thinning, while a large radius can reduce the draw depth, hence not utilising the formability of the material effectively [30]. As a result of these control methods, the flow of material into the die changes, which in turn changes the effect of friction and thus the product quality [43]. This complexity underscores the challenge in achieving highly accurate numerical predictions of product quality in deep drawing. The recommendations of control and design parameters in deep drawing vary significantly across different materials and part geometries, and consequently, research tends to focus on processes designed to react at the onset of failure rather than developing a universal model. Typically, researchers employ numerical models to predict failure; yet, as noted by [44], the accuracy of these predictions is significantly impacted by factors such as anisotropic material behaviour, friction coefficients, material characterisation, and the selection of element types in the simulation. Hence, physical experiments remain essential to validate the results of numerical models. Amongst all the process and control parameters in deep drawing, the die and punch radii exert the greatest influence on thickness profile, exceeding the impact of other factors by 35% [45]. At the punch radius, the workpiece experiences a positive Gaussian curvature which induces tensile strains, resulting in thinning. In contrast, at the die radius, the workpiece experiences a negative Gaussian curvature, resulting in a deformation close to pure shear.

Evidently, several thickness control mechanisms exist across conventional spinning, incremental sheet forming, and deep drawing. However, the primary reason for thickness changes originates from the profile of the local curvature at the deformation zone. A negative Gaussian curvature in spinning induces deformation in a state close to pure shear [46], while a positive Gaussian curvature in incremental sheet forming [24] and deep drawing induces thinning [8]. The influence between Gaussian curvature at the deformation zone and the resulting thickness change is not well documented for the above processes. However, the above literature has found a strong correlation between tool design parameters and the final thickness profile, suggesting that the explicit integration of curvature-driven strain mechanics into die design could enable universal thickness control strategies.

In folding-shearing, the impact of die design has not yet been investigated. Research has explored how blank design [14] and the beak angle [15] impact the product thickness distribution, but the specific role of the beak shape remains unexplored. As this review of the literature has established that Gaussian curvature is a fundamental determinant of thickness variation in forming processes, this paper aims to understand whether a curved beak, with a non-zero Gaussian curvature, might allow better control of the thickness distribution.

## Process concept and analytical models

**Fig. 2:**
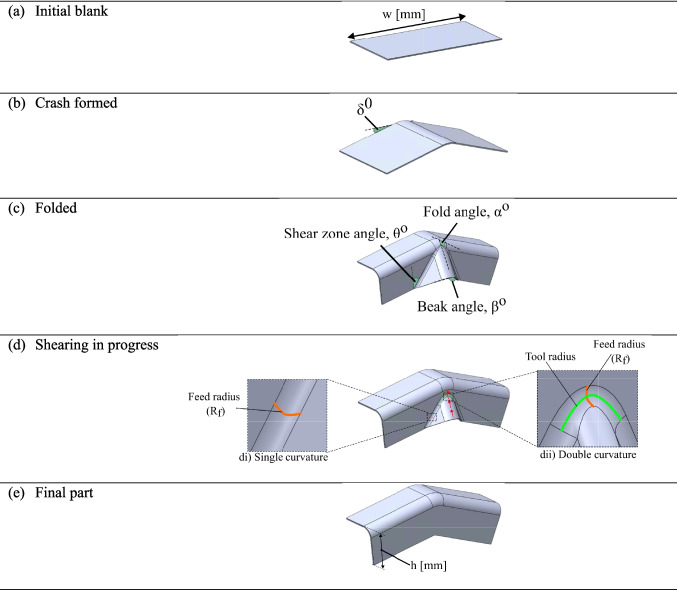
Blank progression: (**a**) flat rectangular blank; (**b**) after crash forming; (**c**) after folded; (**d**) during shearing; (**e**) final target geometry

This paper builds upon the work of [15] and uses the same half of a U-channel part geometry. This approach facilitates a comparative analysis between a flat-faced and a curved beak geometry. Figure 2 demonstrates the subsequent steps required to achieve the target geometry.

A flat rectangular blank of width $w$ (mm) (Fig. 2a), is first crash formed by an angle of $\delta$ (deg) to obtain Fig. 2b. Subsequently, the part is bent by 90° to get to obtain Fig. 2c and the excess material is collected in the beak. Once the beaks are formed, a set of tools grip the material within the beak and move vertically downwards while the material bends and unbends through the feed radius (${R}_{f}$ in Fig. 2d) to form the flat sidewall of height, $h$ (mm) (Fig. 2e). In the deformation zone, the material experiences two different curvatures: towards the edge, the material experiences a single curvature (Fig. 2d(i)), while at the centre, the material experiences a negative Gaussian curvature (Fig. 2d(ii)). A previous work examining the process in Fig. 2 [15] revealed that a deformation close to pure shear was only seen at the centre of the beak. This paper investigates whether modifying the beak geometry can further enhance control over the resulting thickness distribution.

### Geometry of curved beaks

**Fig. 3:**
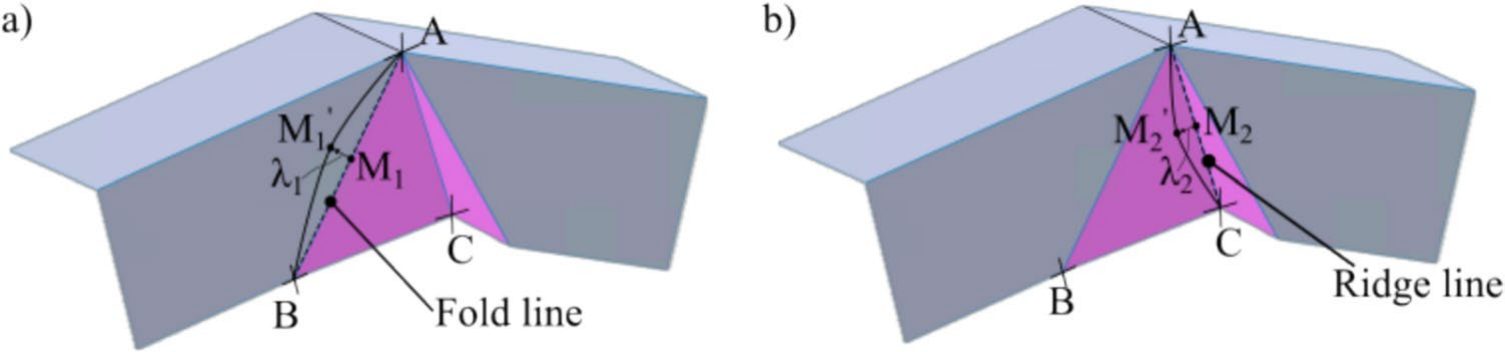
Adding curvature to (**a**) fold line and (**b**) ridge line

Insights from the literature suggested that introducing a negative Gaussian curvature can enable a deformation closer to pure shear. This raises a question of whether the beak geometry can deliberately be designed to influence the deformation mode. To explore this, this section describes the methodology used to design curved beak geometries. It captures fundamental trigonometric relationships and lays the mathematical foundation for subsequent process analysis. Curvature is introduced by offsetting the midpoint of straight-line segments of a classical flat-faced beak. This can result in a concave or a convex profile depending on the direction of the offset. Figure 3 illustrates this transformation, where the original straight-line segments of the beak, namely AB, BC, and AC, are modified to create curved geometries.

Line BC is formed from the extrusion of line AB along the line AC. Hence, it is only necessary to control the line AB and the line AC to define the overall shape of the beak. Henceforth, line AB is called the ‘fold line’ and line AC is called the ‘ridge line’. Figure 3a shows that the points A, B, and ${M}_{1}$ lie on the same line and are in the same plane. ${M}_{1}$ is the midpoint of the fold line and can be offset by a perpendicular distance of ${\lambda }_{1}$, until it reaches a new point ${M}_{1}{\prime}$. Points ($A{M}_{1}{\prime}B$) now sit on a circular arc and form the new convex curved fold line. Similarly, points A, ${M}_{2}$, and C lie on the ridge line (Fig. 3b) and are on the same plane. ${M}_{2}$ is the midpoint of AC and can be offset by a perpendicular distance of ${\lambda }_{2}$ to reach a new point ${M}_{2}{\prime}$. Points ($A{M}_{2}{\prime}C$) now sit on a circular arc and form the new concave ridge line. ${\lambda }_{1}$ and ${\lambda }_{2}$ can be offset in the opposite direction to obtain a concave or a convex curvature, respectively. The maximum allowable offsets (${{\lambda }_{1}, \lambda }_{2}$) are defined by the tangency limits made by the circular arcs to points A, B, or C, such that each arc does not surpass the existing boundary of the part or penetrate the surface in any direction. Table 1 summarises the resulting trigonometric functions that define the maximum offset for both the ridge and fold line at A, B, or C as:

**Table 1:** Trigonometric functions to define maximum offsets to satisfy tangency condition

Line	Point	Convex	Concave
Fold line,${\lambda }_{1}$	A	$\frac{h(1-sin\theta )}{2{\mathit{cos}}^{2}\theta }$	$\frac{h(1-\text{cos}(\theta -\delta )}{2\text{cos}\theta \text{sin}(\theta -\delta )}$
	B	$\frac{h(1-cos\theta )}{2cos\theta sin\theta }$	$\frac{h(1-sin\theta )}{2{\mathit{cos}}^{2}\theta }$
Ridge line,${\lambda }_{2}$	A	$\frac{h(1-cos\theta )}{2{\text{sin}}^{2}\theta }$	$\frac{h(1-sin\theta )}{2cos\theta }$
	C	$\frac{h(1-sin\theta )}{2cos\theta }$	$\frac{h(1-cos\theta )}{2{\text{sin}}^{2}\theta }$

Caution must be taken as the design is based on the minimum value of the offsets for each point on the corresponding line. For example, if the fold line is intended to be convex, then the minimum ${\lambda }_{1}$ from points A or B must be considered, as shown in Eq. 1. If the offset exceeds this minimum value, then the tool geometry is no longer tangent to one of the axes at A or B and this will result in uneven tool geometries.

1 $${\lambda }_{{1}_{convex,max}}=\text{min}\left(\frac{h(1-sin\theta )}{2\;{\mathit{cos}}^{2}\;\theta }, \frac{h(1-cos\theta )}{2cos\theta\; sin\theta }\right)$$

**Fig. 4:**
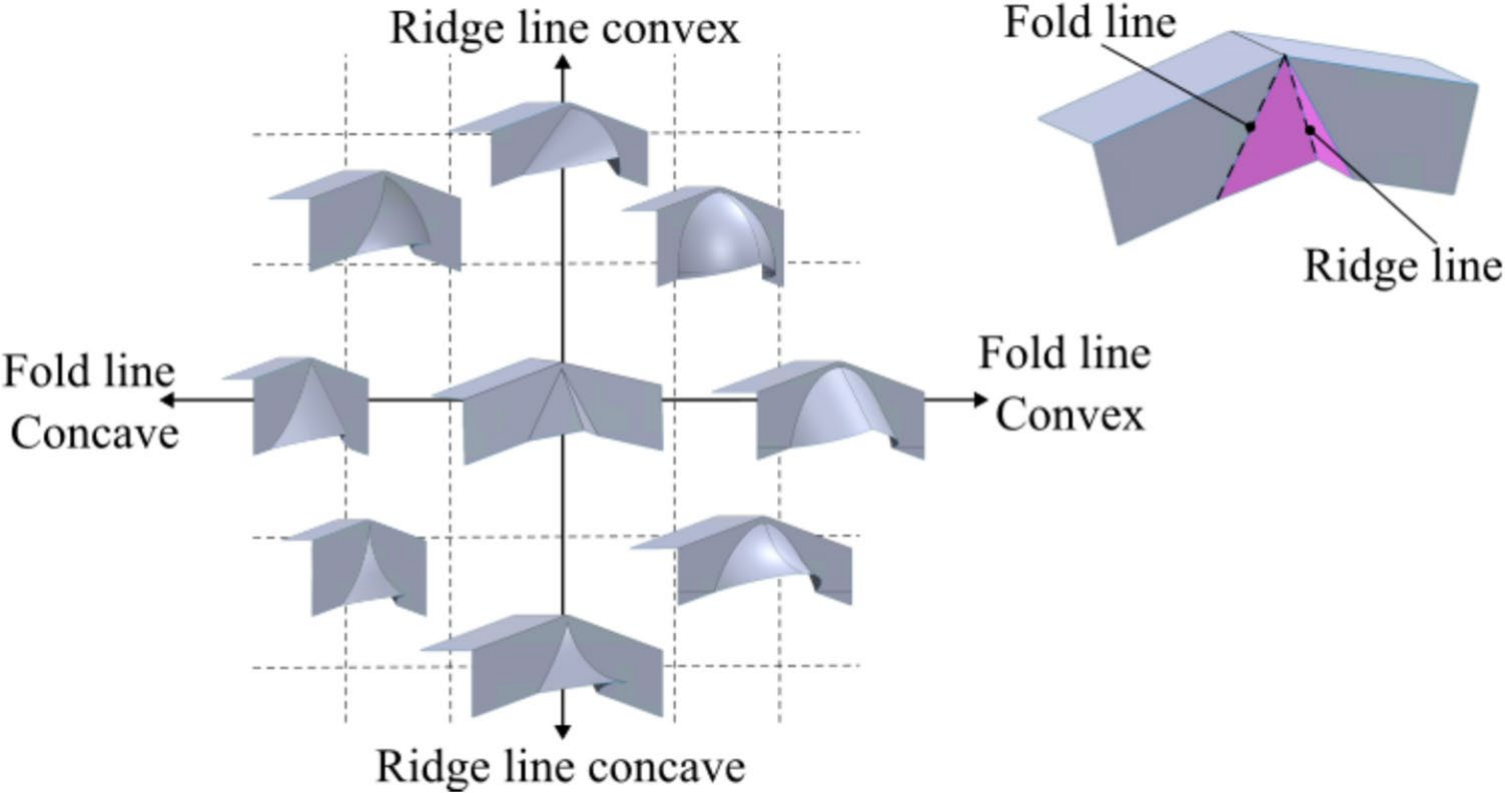
Design space while considering curvature along the fold and the ridge line

With the maximum displacements defined, a novel design space can be mapped as shown in Fig. 4.

The graph plots the fold line along the x-axis and the ridge line along the y-axis. Since a concave surface bends inwards, it is in the negative region of the axis, and a convex offset is on the positive region of the axis. The flat-faced beak from [15] is seen at the centre (0,0), while the other coordinates represent a curved beak. Progressing along the x-axis, from left to right, the fold line is initially concave and gradually becomes convex, while the ridge remains linear. Progressing along the y-axis, from top to bottom, the ridge line is initially convex and gradually becomes concave, while the fold line remains linear. In each corresponding quadrant, four distinct tool combinations are identified, where a curvature is seen along both the lines. A consequence of using a curved beak is the loss of developability. Unlike flat-faced beaks that result in a developed folded surface, a curved beak introduces in-plane stretching that will influence the strain distribution. The impact of this approach will be discussed later.

**Fig. 5:**
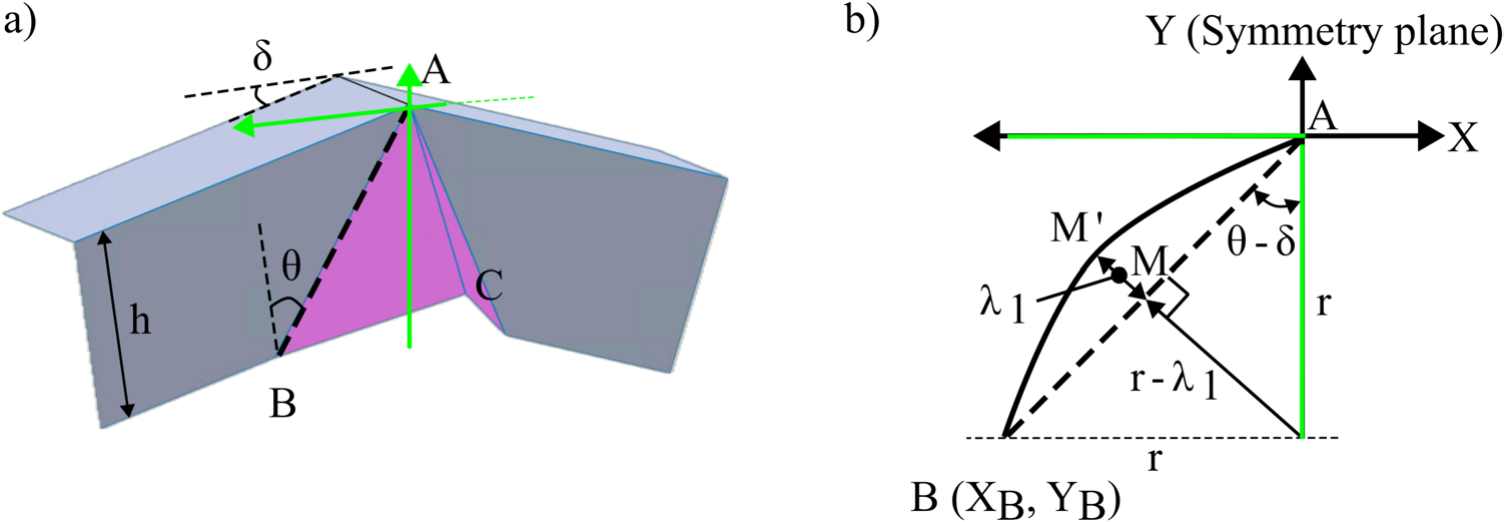
(**a**) Parametric view with key parameters. (**b**) Offsetting line AB by ${\lambda }_{1}$ to introduce a curvature

Developing a mathematical understanding of the curvature is essential as it will directly inform the resulting strains imposed by a tool design. Figure 5a highlights the key parameters used to define the curvature along the fold line, and Fig. 5a presents a parametric view of the folded shape using an origami model with points A, B, and C labelled. The points A and B lie on the same plane, which is on the sidewall, and the key process parameters are shown in Fig. 5b.

Curvature is added to the fold line by offsetting the point M to M’ and it is known that points A, M’, and B lie on a circular arc and take the form of an equation of a circle as:

2 $${\left(x-{x}_{{0}{\prime}}\right)}^{2}+{\left(y-{y}_{{0}{\prime}}\right)}^{2}={\rho }_{1}^{2}$$

where, (${x}_{{0}{\prime}},{y}_{{0}{\prime}}$) is the co-ordinate of the centre of the circular arc and ${\rho }_{1}$ is the radius.

Since A lies at ($0,{y}_{A}$) and B lies at (${x}_{B},0$), it is assumed that the circle’s centre lies somewhere along the perpendicular bisector of line AB. The distance from the centre to the points of A and B must both be equal to ${\rho }_{1}$ and given that the curvature is introduced by offsetting the fold line by ${\lambda }_{1}$, the centre moves by ${\lambda }_{1}$ in the direction normal to the fold. Assuming symmetry in the x-direction, the centre of the circular is found as:

3 $$\left({x}_{{0}{\prime}},{y}_{{0}{\prime}}\right)=(0,{y}_{A}-({\rho }_{1}-{\lambda }_{1}))$$

The X and Y co-ordinates of point B can be found through trigonometric functions as:

4 $${x}_{B}=\frac{h}{\text{cos}(\theta )}\text{sin}(\theta -\delta )$$

Point B lies on the X axis and the x coordinate of point B (${x}_{B}$) can be found through trigonometric functions as:

5 $$\begin{array}{c}{x}_{B}=\frac{h}{\text{cos}(\theta )}\text{sin}(\theta -\delta )\\ {y}_{B}=\frac{h}{\text{cos}(\theta )}\text{cos}(\theta -\delta )\end{array}$$

Since point B lies on the circular arc, the radius of the arc can be found by substituting Eq. 5 and Eq. 4 into Eq. 3, resulting in:

6 $${{(x}_{B}-{x}_{{0}{\prime}})}^{2}+{\left({y}_{B}-\left[{y}_{A}-\left({\rho }_{1}-{\lambda }_{1}\right)\right]\right)}^{2}={\rho }_{1}^{2}$$

The radius of the circular arc is found after simplifying Eq. 6 as:

7 $${\rho }_{1}=\frac{{\lambda }_{1}}{2}+\frac{1}{8{\lambda }_{1}}{\left(\frac{h}{\text{cos}\left(\theta \right)}\right)}^{2}\left\{{\text{sin}}^{2}\left(\theta -\delta \right)+{\text{cos}}^{2}\left(\theta -\delta \right)\right\}$$

Since ${\text{sin}}^{2}\left(x\right)+{\text{cos}}^{2}\left(x\right)=1$, the resulting radius of the circular arc is:

8 $${\rho }_{1}=\frac{{\lambda }_{1}}{2}+\frac{1}{8{\lambda }_{1}}{\left(\frac{h}{\text{cos}\left(\theta \right)}\right)}^{2}$$

From metal forming literature, the curvature ($\kappa$) due to a radius ($\rho$) can be found as:

9 $$\kappa =\frac{1}{\rho }$$

Hence, the curvature due to the fold line (${\kappa }_{1}$) is obtained as:

10 $${\kappa }_{1}(h,\theta ,\delta )=\frac{1}{{\rho }_{1}}=\frac{1}{\left(\frac{{\lambda }_{1}}{2}+\frac{1}{8{\lambda }_{1}}{\left(\frac{h}{\text{cos}\left(\theta \right)}\right)}^{2}\right)}$$

Depending on whether the fold line has a convex or concave curvature, ${\lambda }_{1}$ can be substituted from Table 1 into Eq. 10. Hence, the resulting curvature of the fold line is a function of the part height, the shear zone angle, and the crash angle. Using the same logic, the curvature of the ridge line (${\kappa }_{2}$) can be established:

11 $${\kappa }_{2}(h,\theta ,\delta )=\frac{1}{{\rho }_{2}}=\frac{1}{\left(\frac{{\lambda }_{2}}{2}+\frac{1}{8{\lambda }_{2}}{\left(h\right)}^{2}\right)}$$

The resulting curvature due to the ridge line is a function of the part height, the shear zone angle, and the crash angle. These resulting curvatures are useful to define the thickening or thinning and are discussed next.

### Analytical model of thickness

In folding–shearing, the workpiece is expected to undergo thinning at the apex and a thickness change along the sidewall. To capture this behaviour, a general analytical model is developed as a function of resulting curvatures, whilst drawing inspiration from the Nakajima test. This is a standardised formability test (ISO 12004–2), where a hemispherical punch deforms a blank until a necking failure is observed [47]. The material’s strain limits are identified and are used to determine the forming limit curve of the respective material [48]. Different variations of the Nakajima test have been explored, where the shape of the blank is modified such that different tensile strains along the directions are captured, enabling deformation modes ranging from uniaxial to biaxial stretching [49]. Notably, a key resemblance between a Nakajima test and folding–shearing with a curved beak is that a thickness change arises from varying curvatures in both orthogonal directions. This similarity forms the basis for extending the principles of a Nakajima thickness model to folding–shearing, where the same underlying concepts are applied to account for curvature-induced thickness changes and are explained below. Once the material passes through a deformation zone, it is assumed that the thickness of the workpiece remains unchanged. Thus, the analysis stated below is applicable through the stroke of the process and the model is developed solely based on the bending strains that arise from the curvatures.

The model assumes plastic incompressibility, where the in-plane strains lead to a corresponding thickness reduction [8]. Assuming volume constancy, the thickness strain in terms of the bending strains in two orthogonal directions is given as:

12 $${\varepsilon }_{t}=-({\varepsilon }_{b1}+{\varepsilon }_{b2})$$

where, ${\varepsilon }_{t}$ is the thickness strain, ${\varepsilon }_{b1}$ is the bending strain in one direction, and ${\varepsilon }_{b2}$ is the bending strain in the other.

The bending strain (${\varepsilon }_{b}$) is found by considering the natural logarithm of the new length (${l}_{1}$) over the original length (${l}_{0}$) and is given as [8]:

13 $${\varepsilon }_{b}=\mathit{ln}\left(\frac{{l}_{1}}{{l}_{0}}\right)$$

As the workpiece is bent over a radius of $\rho$, by an angle of $\phi$, the length of the neutral axis is given as:

14 $${l}_{0}=\phi \rho$$

Consecutively, the new length is calculated by considering a point at ‘$y$’ distance away from the neutral axis and is given as:

15 $${l}_{1}=\phi (\rho +y)$$

Hence, the bending strain is found by substituting Eq. 14 and Eq. 13 into Eq. 12 to obtain:

16 $${\varepsilon }_{b}=\mathit{ln}\left(\frac{{l}_{1}}{{l}_{0}}\right)=\mathit{ln}\left(\frac{\phi (\rho +y)}{\phi \rho }\right)= \mathit{ln}\left(\frac{\rho \phi \left(1+\frac{y}{\rho }\right)}{\rho \phi }\right)=\mathit{ln}\left(1+\frac{y}{\rho }\right)$$

The natural logarithm (Eq. 16) can be simplified by its first-order Taylor series expansion to Eq. 17 as [8]:

17 $${\varepsilon }_{b}\cong \left(\frac{y}{\rho }\right)$$

For a symmetrical bending, the change in thickness ($\Delta t$) that occurs due to the bending strains can be found by integrating the resulting thickness strains across the initial sheet thickness as:

18 $$\Delta t={\int }_{-{t}_{0}/2}^{{t}_{0}/2}-({\varepsilon }_{b1}+{\varepsilon }_{b2})dy$$

Since the ratio of the part radius to material thickness is expected to be less than 5, it is assumed that the material experiences a fully plastic bending [8], with the neutral axis shifted to the inner edge of the workpiece. Thereby, the thickness change can be found by considering an asymmetrical strain distribution as:

19 $$\Delta t={\int }_{0}^{{t}_{0}}-\left(\frac{y}{{\rho }_{1}}+\frac{y}{{\rho }_{2}}\right)dy=-\frac{{t}_{0}^{2}}{2}\left(\frac{1}{{\rho }_{1}}+\frac{1}{{\rho }_{2}}\right)$$

Equation 19 can be simplified to find the final thickness (${t}_{f}$) as a result of the two curvatures as:

20 $${t}_{f}={\text{t}}_{0}+\Delta t={t}_{0}-\frac{{t}_{0}^{2}}{2}\left(\frac{1}{{\rho }_{1}}+\frac{1}{{\rho }_{2}}\right)$$

Equation 20 can be simplified by considering the curvature (Eq. 10) in both the directions as ${\kappa }_{1}$ and ${\kappa }_{2}$ as:

21 $${\text{t}}_{\text{f}}={\text{t}}_{0}+\Delta t={t}_{0}-\frac{{t}_{0}^{2}}{2}\left({\kappa }_{1}+{\kappa }_{2}\right)$$

${\kappa }_{1}$ and ${\kappa }_{2}$ must be calculated with caution as the resulting curvature can be either positive or negative if the radius is convex or concave, respectively. As postulated by Gaussian curvature literature, in the case of pure shear, the curvature along one direction is positive, while the curvature along the other direction is negative. If these radii are equal in magnitude, then -${\kappa }_{1}={\kappa }_{2}$, and Eq. 21 simplifies to show that ${t}_{f}={t}_{0}$, indicating pure shear deformation. Equation 21 is revealing as it shows that the final thickness is only dependent on the curvatures and the initial thickness. This demonstrates that in a pure shear deformation, if the curvatures are balanced, the thickness remains unchanged, making curvature control the key to minimising thickness variation.

**Fig. 6:**
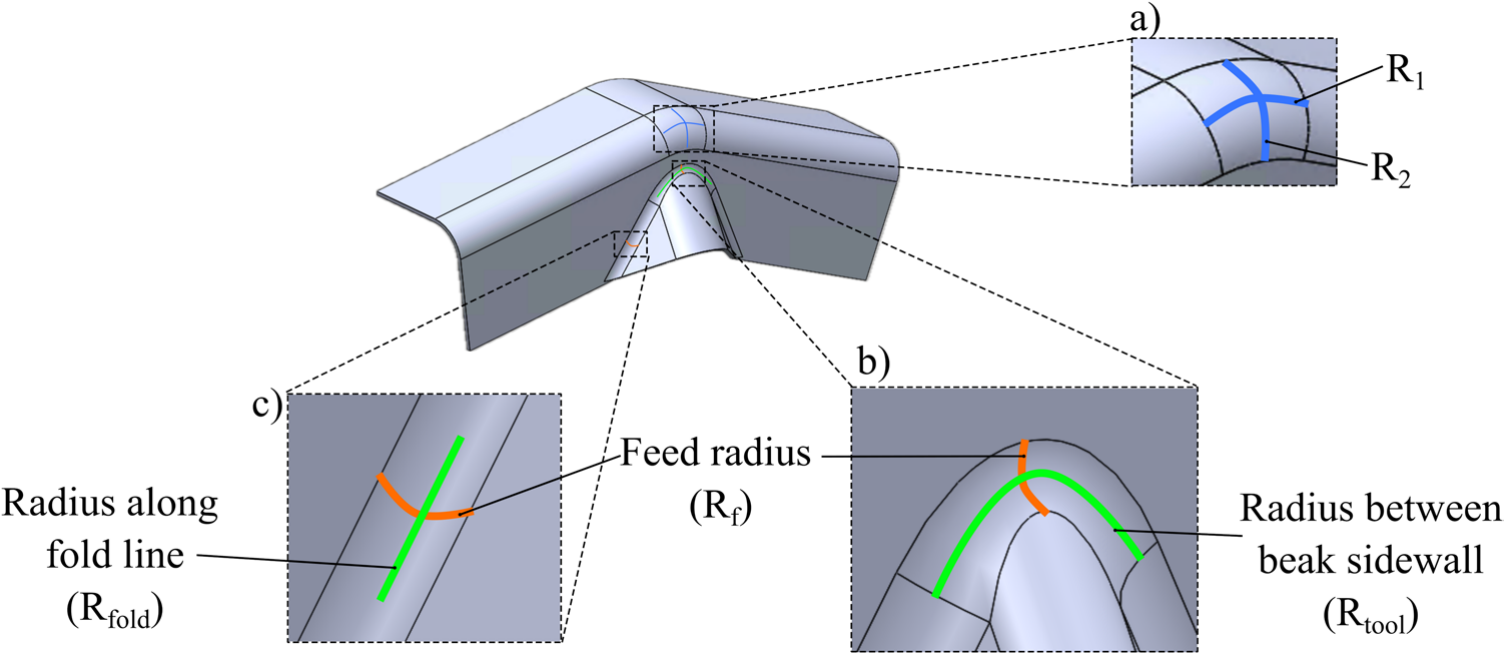
Radius in different regions resulting in thickness change: (**a**) convex ${R}_{1}$ and convex ${R}_{2}$ at the apex; (**b**) concave ${R}_{f}$ and convex ${R}_{tool}$; (**c**) concave ${R}_{f}$ and ${R}_{fold}$ (figure shows ${R}_{fold}=\infty$)

Analogously in folding–shearing, the thickness change occurs in critical regions due to curvature. Since the workpiece experiences curvatures only in the deformation zone, it is assumed that the resulting thickness remains constant throughout the rest of the stroke once the workpiece passes through this zone. It is assumed that the plane sections remain plane before and after the deformation process, indicating that there is no through-thickness shear deformation. Equation 20 is now extended to describe thickness change in three critical regions: apex (Fig. 6a), centre of the sidewall (Fig. 6b), and the edge of the beak in the sidewall (Fig. 6c).

As shown in Fig. 6, each region is characterised by a unique combination of principal radii, leading to distinct strain distributions and corresponding thickness changes. These three regions are critical to the process as they represent the most probable location of failure of thinning or thickening. In contrast, other regions primarily undergo conventional bending, where no significant thickness changes are expected. By substituting the principal radii into Eq. 20, the resulting thickness can be calculated explicitly for metal deforming at that specific region.

At the apex (Fig. 6a), the workpiece experiences a thickness change due to two convex radii: ${R}_{1}$ and ${R}_{2}$. This results in the final thickness of the workpiece at the apex (${t}_{\text{f}\_\text{apex}}$) as shown in Eq. 22.

22 $${\text{t}}_{\text{f}\_\text{apex}}={t}_{0}-\frac{{t}_{0}^{2}}{2}\left(\frac{1}{{R}_{1}}+\frac{1}{{R}_{2}}\right)$$

Equation 22 predicts thinning of the blank due to the two convex radii and is consistent with metal forming mechanics where a material is stretched biaxially over two cove surfaces. At the centre of the beak (Fig. 6b), the workpiece experiences a thickness change as it bends and unbends via the concave feed radius (${R}_{f}$) and experiences stretching due to the convex radius between the walls of the beak (${R}_{tool}$) as shown in Eq. 23. A key observation from Eq. 24 is that ${R}_{tool}$ must have a convex curvature to balance the bending strains induced by ${R}_{f}$.

23 $${t}_{\text{f}\_\text{sidewall}\_\text{centre}}={t}_{0}-\frac{{t}_{0}^{2}}{2}\left(\frac{-2}{{R}_{f}}+\frac{1}{{R}_{tool}}\right)$$

At the edge of the beak (Fig. 6c), the material experiences a thickness change as it bends and unbends via the concave feed radius (${R}_{f}$) and experiences a thickness change due to the curvature along the fold line (${R}_{fold}$) as shown in Eq. 24.

24 $${\text{t}}_{\text{f}\_\text{sidewall}}={t}_{0}-\frac{{t}_{0}^{2}}{2}\left(\frac{-2}{{R}_{f}}+\frac{1}{{R}_{fold}}\right)$$

A key observation from Eq. 23 and Eq. 24 is that ${R}_{tool}$ or ${R}_{fold}$ must have a convex curvature to balance the bending strains induced by ${R}_{f}$. When these curvatures are balanced, the deformation approaches a state of pure shear as the material experiences equal tensile and compressive strains. The general analytical model of thickness is demonstrated to be applicable to both thinning and thickening and will be validated through both physical trials and numerical models. The model assumes that the workpiece thickness remains unchanged after undergoing deformation. Specifically, once the part has undergone bending or shearing, its thickness is considered constant throughout subsequent stages. This simplification allows for a more straightforward analysis of the process while still capturing the essential mechanics of the folding–shearing operation. The following section introduces the numerical model setup and validation.

## Numerical investigation

A numerical model is developed to evaluate the impact of beak geometries on process outcomes such as thickness variation and springback. This model enables a systematic comparison of different beak geometries across various aspect ratios and material parameters. The objective of this section is to identify an optimal curved beak design for subsequent physical validation.

### Model setup

**Fig. 7:**
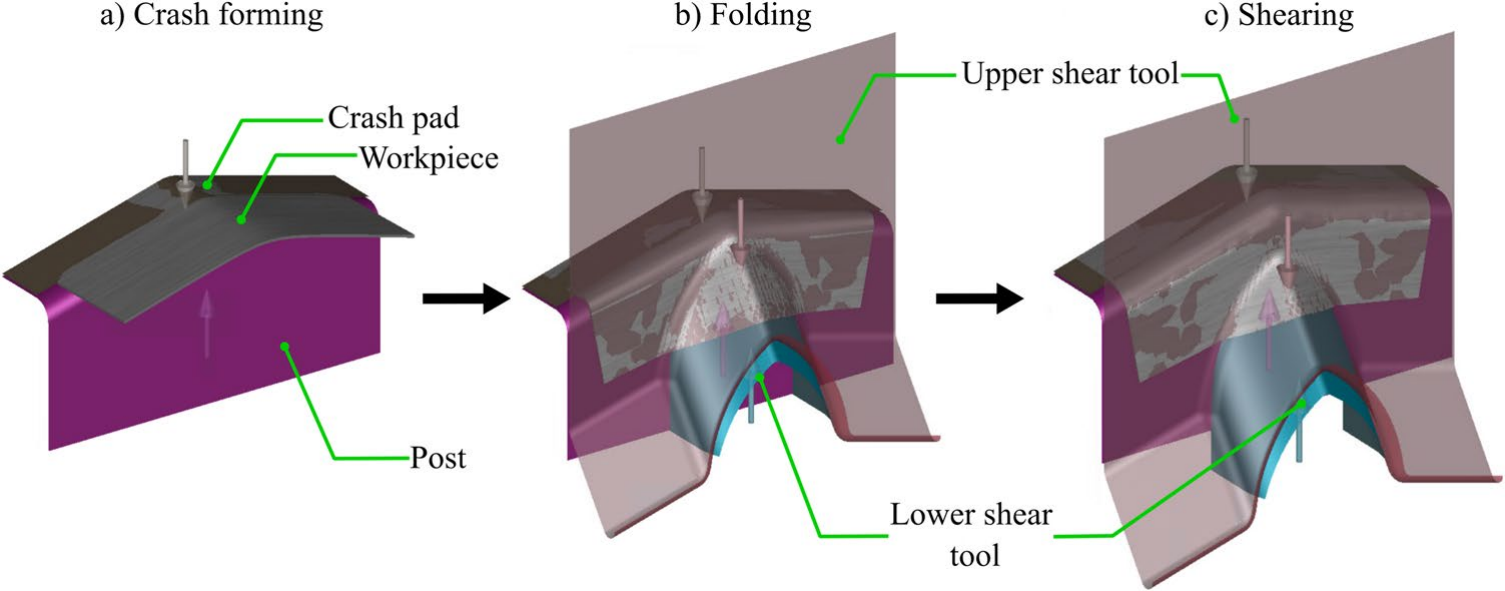
Numerical model setup: (**a**) crash forming, (**b**) folding, (**c**) shearing

The numerical model was developed using an implicit time integration approach in Autoform R11. This approach solves the static equilibrium equations using a Newton–Raphson scheme, where internal and external forces are balanced at each time increment, and material integration is handled through standard return mapping algorithms built into the software. The geometry of the tool surfaces was modelled in CAD software and then imported as step files, while the rectangular blank was directly sketched in Autoform. The production line was set up with a ‘draw’ operation and the tools were located on the ram or the bed. In the first stage (Fig. 7a), a rigid crash pad moved vertically to deform the workpiece on a rigid post until it reached the required crash angle. The crash pad is then locked in position to ensure that the tool does not lift up during the remainder of the operation. In the next stage (Fig. 7b), the upper shear tool was moved vertically downwards, which folded the workpiece over the post radius until the beak was formed. In the final stage (Fig. 7c), the upper and the lower shear tools were moved vertically together, and the beak was deformed to form the flat sidewall.

The workpiece was modelled using thick-shell elements, and automatic mesh refinement was enabled with seven refinement levels. A mesh sensitivity study was conducted by varying two key parameters: (a) the in-plane mesh size, and (b) the number of integration points through the sheet thickness. The mesh size was systematically reduced using the following element sizes: 0.2 mm, 0.15 mm, 0.1 mm, 0.05 mm, and 0.025 mm. For each mesh size, a simulation was run using either five or 11 integration points through the sheet thickness. This combination of parameters resulted in a total of 10 potential mesh configurations. The predicted thickness from each simulation was compared against experimental measurements, as reported by Arora et al. [15]. Across all simulations, the maximum deviation was 12.5% for the coarsest mesh, and the minimum deviation was approximately 5% for the finest mesh. Amongst these, the mesh size of 0.05 mm yielded results to within 6.25% of the experimental data and was considered sufficiently accurate as it ensured at least five elements across the smallest radius. Increasing the number of integration points from five to 11 provided a marginal improvement of 2.7%, in line with findings from [50]. However, this increase came with an increased computation time: a mesh size of 0.05 mm with 11 integration points was 1 min 32 s longer than the corresponding simulation with five integration points. This was still considered efficient, and to balance computation time and model accuracy, a mesh of 0.05-mm element size was used with 11 integration points for all further simulations. An elastic–plastic material model was used from the supplier database, which was described by the BBC2005 anisotropic yield criterion and the Swift work-hardening law. Finally, a Coulomb-friction model was assumed with the coefficient of friction of 0.15. Each simulation took approximately 5 min on a notebook with an Intel Core i5 processor.

### Design of test

**Fig. 8:**
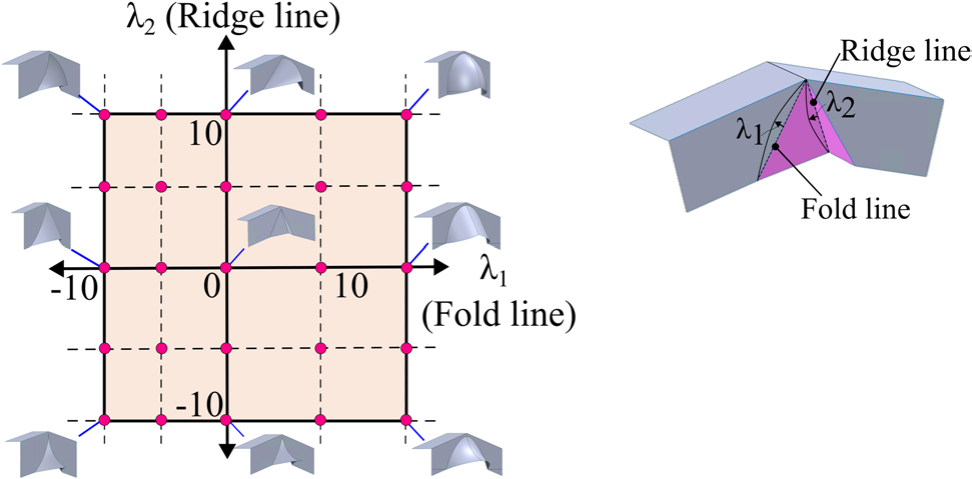
25 unique tool designs for a shear zone angle of $45^\circ$ and crash angle of $15^\circ$

The design methodology involved selecting five levels of curvature for both the fold and the ridge line: the maximum concave offset, half of the maximum concave offset, zero offset, half of the maximum convex offset, and maximum convex offset. This resulted in a total of 25 tool designs across the design space, as shown in Fig. 8. The intersection of each line represents a unique tool design, with the tool at the origin corresponding to flat-faced beaks from [15] while all other designs represent a curved beak.

A crash angle of 15° and a shear zone angle of 45° were chosen based on previous research identifying this as the threshold between the limits of thinning and thickening [15]. This design parameter lies at the lower boundary of the process operating window and presents an opportunity to expand the overall window.

**Fig. 9:**
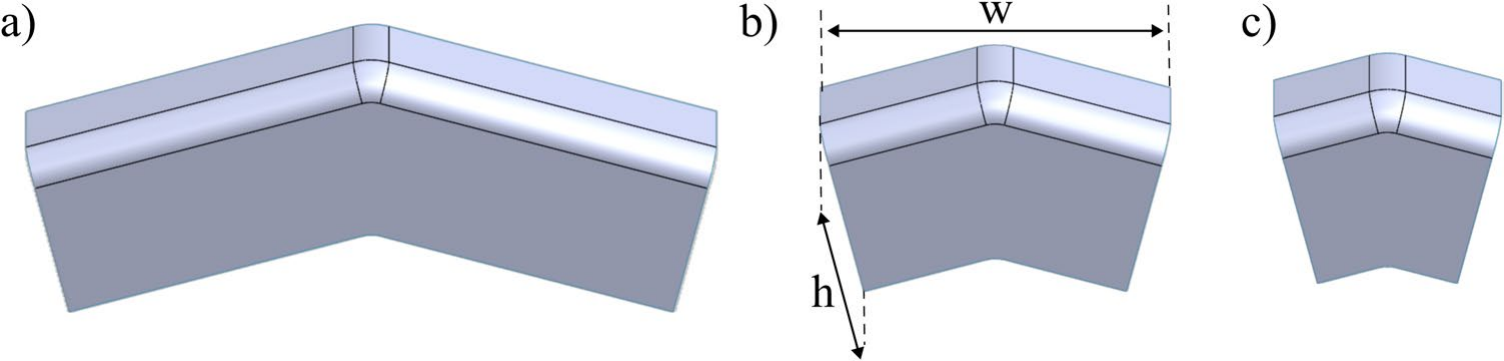
Varying part ratios: (**a**) 0.25, (**b**) 0.5, (**c**) 0.75

The investigation explores the range of parts that can be formed across the design space using different material parameters. A key parameter defining the final part geometry is the aspect ratio, which is the ratio of the part height to the part width (Fig. 9). To maintain consistency with previous research using straight-edged beaks [15], the same materials were used to ensure comparability: two grades of aluminium alloys AA1050-H14 and AA5083-H111 were tested. AA5083 was chosen in this paper as it is commonly used in automotive and marine applications and offers a good balance between strength, weldability, and corrosion resistance. The resulting parameters used are summarised in Table 2.

**Table 2:** Design of experiments parameters

Number of tool designs	25
Material	AA1050-H14, AA5083-H111
Crash angle	$15^\circ$
Shear zone angle	$45^\circ$
Aspect ratio	$0.25, 0.5, 0.75$
Part thickness	$1.5$ mm

### Results and discussion

Building on the experimental design outlined in Sect. 4.2, this section examines the influence of curved beak geometries on final part results, specifically thinning, thickening, and springback. A total of 150 numerical trials were conducted across three aspect ratios, evaluating 25 tools designs and two materials to assess the impact of tool geometry. For a part to be considered successful, the thinning and thickening must be lower than 10% and the springback, after complete tool removal, must be lower than 1 mm. Higher levels of thinning can result in necking, which may lead to tearing. Conversely, excessively thickening can cause the workpiece to lock between the tools, leading to a pre-mature tear. A springback tolerance of $\pm 1$ mm is considered acceptable in industrial applications, as such deviations can be typically corrected through a re-strike. However, re-striking is a highly non-linear process, and any greater springback leads to worsened dimensional accuracy, thus increasing complexity for the downstream restrike operation.

**Fig. 10:**
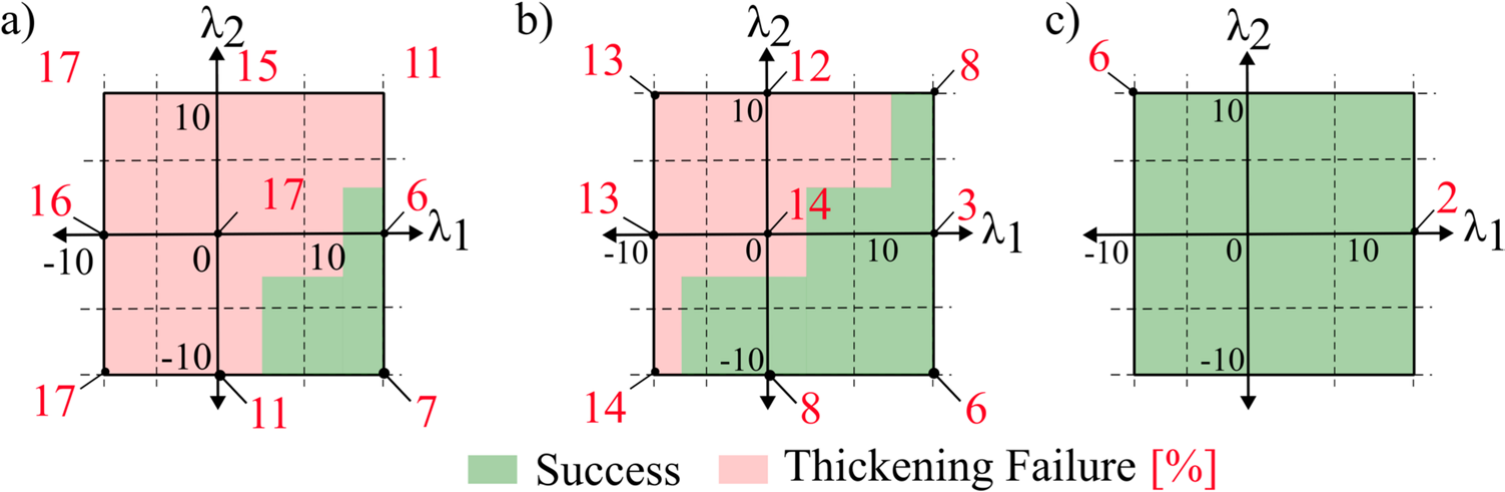
Window showing failure limits using AA1050 across 25 tool designs using aspect ratios of (**a**) 0.25, (**b**) 0.5, and (**c**) 0.75. Relevant thickening values are shown in red

Figure 10 plots the success and failure across the design space for the three aspect ratios using AA1050-H14. If the part was successful using a respective tool, it is shown in green, and if the part has failed due to thickening, it is shown in red. In these plots, the x-axis represents a curvature along the fold line, the y-axis represents a curvature along the ridge line, and the origin represents a flat-faced beak. The only failure mode observed using AA1050 across all the aspect ratios was thickening, where the relevant thickening percentages are shown in red.

A clear trend emerges where the number of successful tools (green region) gradually increases, and eventually all tools are successful from an aspect ratio of 0.75. This is associated with a maximum observable thickening of 6% compared to 17% (for aspect ratio of 0.25 in Fig. 10a). The thickening results are found to be more sensitive to the curvature variation across the fold line (x-axis) rather than the curvature across the ridge line (y-axis). These trends can be attributed to the strain interactions at the deformation zone. Generally, the tools’ designs in quadrants 1, 2, and 3 induced worse thickening strains and can be explained by considering the curvatures at the deformation zone. Along the x-axis, the primary deformation is governed by two curvatures: the concave feed radius and the fold line radius, which may be convex or concave. On the left side of the plots, both the fold line and the feed radius are concave. This results in compressive strains along both the orthogonal planes, and this compounding effect worsens with increased concavity of the fold line. Conversely, on the right side, a convex fold line induces tensile strains, which counteract the compressive strains from the feed radius. Along the y-axis, the curvature influences the thickness at the beak before the workpiece passes through the feed radius. Consequently, its effect is less pronounced than that of the fold line curvature. A convex ridge stretches the material in the beak and reduces the thickness and results in a smaller thickness increase during deformation. However, at higher aspect ratios, this effect becomes negligible.

**Fig. 11:**
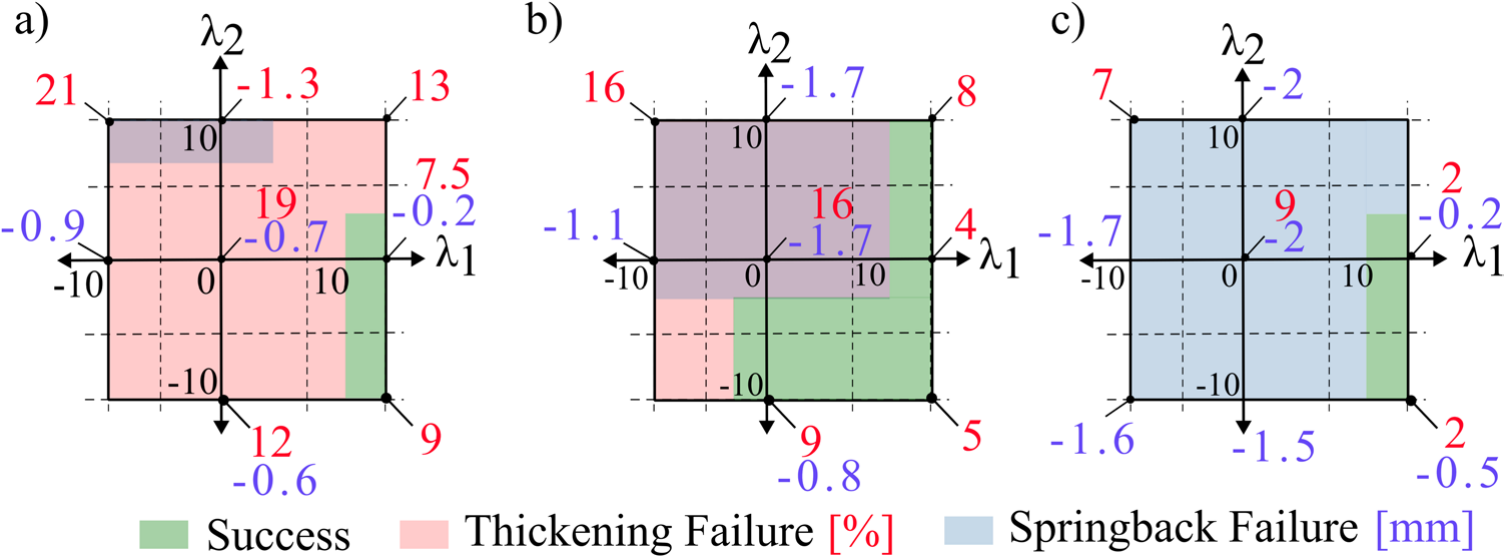
Window showing failure limits using AA5083 across 25 tool designs using aspect ratios of (**a**) 0.25, (**b**) 0.5, and (**c**) 0.75. Relevant thickening and springback values are shown in red and blue

Figure 11 plots the success or failure across the design space for the three aspect ratios using AA5083-H111, and as with Fig. 10, successful tools are shown in green. AA5083 experienced an additional failure mode of springback, and the normal displacement due to springback is shown in blue, while the thickening percentage is shown in red.

Similar to AA1050, the thickening failure remains the dominant failure mode at low aspect ratios and is seen to become less critical at higher aspect ratios. However, the maximum observed thickening values are generally higher compared to that of AA1050. This increase in thickening aligns with previous folding–shearing literature, as metals with higher yield stresses experience a lower elongation before failure, leading to strain concentrations during forming and increased thickening [14], 15. Conversely, the number of tools that result in springback failure increases with aspect ratios. As the aspect ratio increases, the part height relative to the part width is now greater, which amplifies the bending moment and hence leads to a worse springback. The springback failure observed in AA5083 compared to AA1050 is consistent with metal forming mechanics [8], as metals with higher yield stresses exhibit a greater elastic-to-plastic strain ratio during deformation, resulting in higher residual stresses and, consequently, higher springback.

**Fig. 12:**
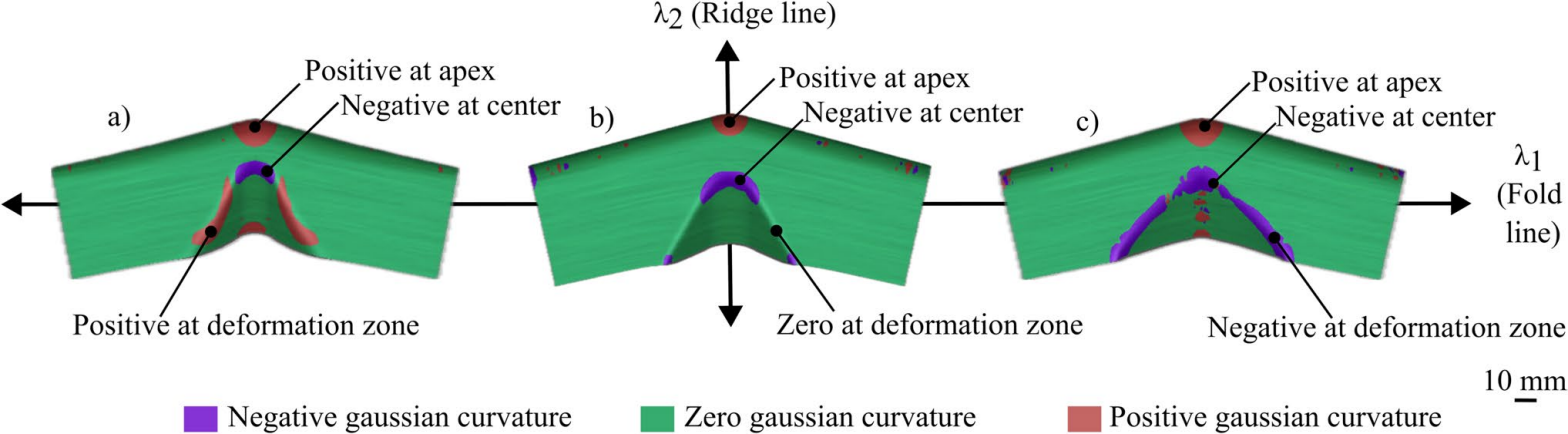
Gaussian curvature due to (**a**) Concave fold line; (**b**) Straight fold line [15]; and (**c**) Convex fold line

Across the range of materials and aspect ratios, the curvature along the fold line was found to have the most significant influence on thickness distribution. This observation aligns with the process mechanics, as the material in the beak deforms along the fold line before forming the flat sidewall. As hypothesised in prior literature, the Gaussian curvature at the deformation zone influences the deformation mode. The remainder of this section validates this theory by comparing three cases of varying Gaussian curvature along the fold line: a tool that induces a positive Gaussian curvature (Fig. 12a), a zeroGaussian curvature (Fig. 12b from [15]), and a negative Gaussian curvature (Fig. 12c). Figure 12 plots the Gaussian curvature at 25% in the shearing stage, where a negative Gaussian curvature is shown in purple, a zero Gaussian curvature is shown in green, and a positive Gaussian curvature is shown in red.

**Fig. 13:**
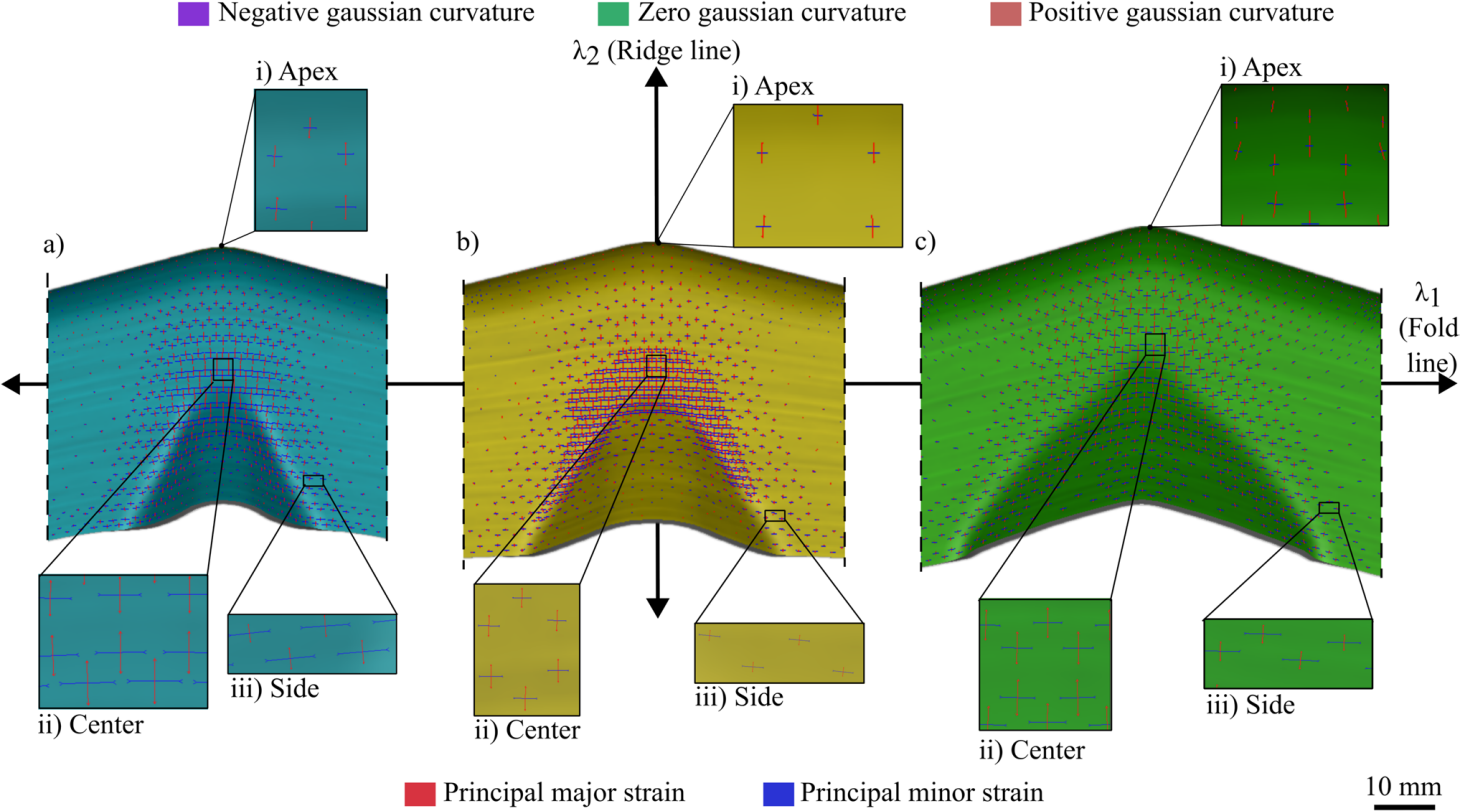
Major and minor principal strains using flat-faced beak versus curved beak at apex, centre*,* and side respectively

A pattern of increasing negative gaussian curvature is observed as the fold line curvature varies from concave (Fig. 12a) to convex (Fig. 12c). Three key regions are identified: the apex, the centre of the beak, and the sides of the beak. Across the three workpieces, the Gaussian curvature at the apex and at the centre of the beak remains consistent, as these regions share the same geometric constraints. The primary difference arises due to varying fold line curvatures, which, when combined with the concave feed radius, result in a shift from positive (Fig. 12a) to zero (Fig. 12b [15]) to negative (Fig. 12c) Gaussian curvatures. The pattern of increasing negative Gaussian curvature is further validated by a corresponding pattern of major and minor strains, which illustrates how the deformation approaches pure shear, as shown in Fig. 13.

Figure 13 maps the principal major strain in red and the minor strains in blue. If the strains are of equal magnitude, hence if the lengths are equal, then the part does not thin or thicken as it experiences equal compression and tension. Across the three key regions, distinct strain distributions are observed. At the apex, the workpiece experiences higher major strains due to a positive Gaussian curvature, arising from convex curvatures in both orthogonal directions, leading to thinning. At the centre of the beak, the strains remain balanced due to negative Gaussian curvature, resulting from the interaction between the convex fold line radius and the concave feed radius, which leads to a minimal thickness change. At the sides of the beak, the strain distribution varies depending on the curvature: in Fig. 13a, the workpiece experiences higher minor strains due to a positive Gaussian curvature, which arises from two concave radii, resulting in *excessive thickening*; in Fig. 13b, the workpiece experiences higher major strains, which arise from a concave feed radius only, resulting in *thickening*. A similar thickening phenomenon is observed in Fig. 13c; however, it is balanced by the major strains induced by the convex fold line radius, which results in a *minimal thickness change*.

This validates the hypothesis that the Gaussian curvature does influence the strain distribution and thereby controls the thickness variation during the forming process. Particularly, a negative Gaussian curvature is crucial in achieving a deformation close to that of pure shear.

## Physical investigation

Building on the numerical analysis from the previous section, it is now possible to design a set of physical trials to validate the assumptions. These experiments assess whether the predicted thickness trends hold across different aspect ratios and material parameters.

### Model setup

**Fig. 14:**
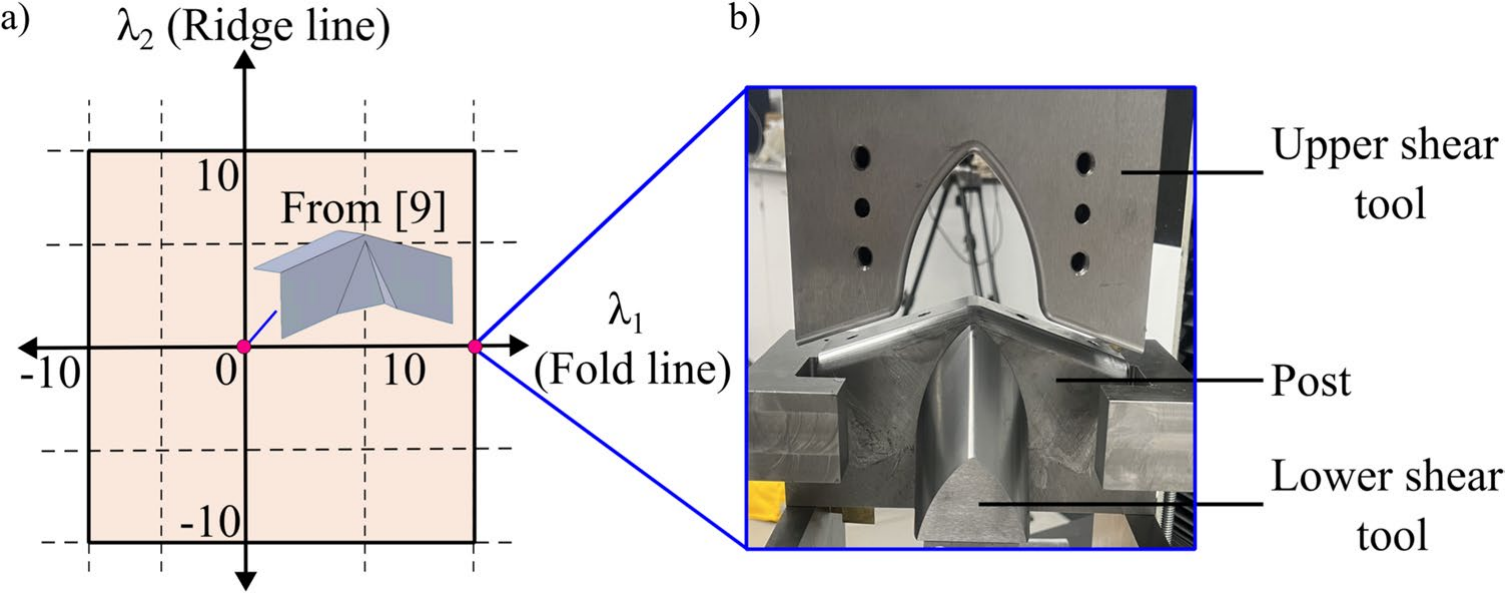
(**a**) Location of the flat-faced beak [15] at origin. (**b**) curved beak tool experimental setup

The numerical investigation showed that the tool with a maximum convex curvature along the fold line produced the optimum thickness result across different aspect ratios and material parameters. Consequently, this tool is chosen for physical prototyping. Figure 14a shows the location of the flat-faced beak at the origin, from [15], and the new curved beak tool on the design space.

Figure 14b depicts the physical tools, where the upper shear tool was secured to the crosshead and the post was fixed at the base of a compression testing machine. The workpiece was clamped and then crash formed over the post until it reached the target crash angle. The upper shear tool was then moved vertically downwards at a constant speed of 1 mm/s until it closed onto the lower shear tool. In the next stage, the upper shear tool was bolted onto the lower shear tool, and both the tools were moved vertically downwards at a constant speed of 1 mm/s. The process continued until the end of the stroke. Following each trial, the force and thickness measurements were recorded. The force data was captured by the load cell that was integrated in the compression testing machine, and the thickness measurements were obtained using an ultrasonic precision thickness gauge.

### Design of test

A total of six physical trials were conducted across two material parameters and three aspect ratios. The primary focus of the physical validation was to assess the three underlying assumptions made in the numerical and analytical models. These assumptions include (i) the thickness change at the apex must remain constant at every aspect ratio; (ii) the material properties should not influence the resulting thickness distribution; and (iii) the thickness of the workpiece remains unchanged once it has passed through the deformation zone. The first and second assumptions can be validated through experimentation with the parameters summarised in Table 3.

**Table 3:** Variables for physical validation

Number of tool designs	2
Material	AA1050-H14, AA5083-H111
Crash angle	$15^\circ$
Shear zone angle	$45^\circ$
Aspect ratio	$0.25, 0.5, 0.75$
Part thickness	$1.5$ mm

**Fig. 15:**
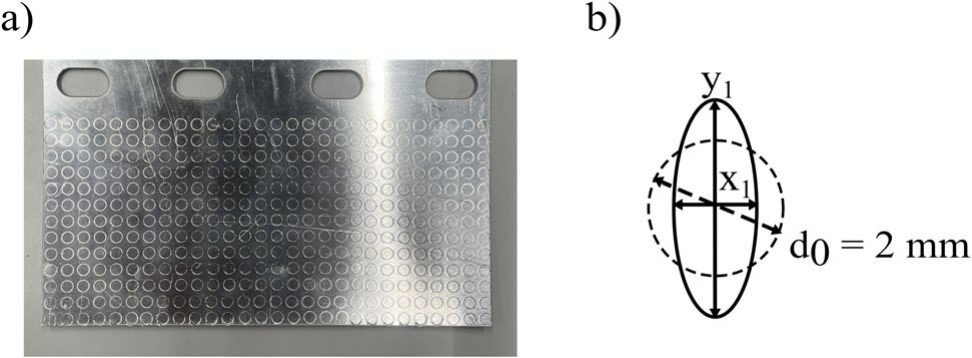
Physical validation of major and minor principal strains

The blanks were cut using a water jet cutter, with their orientation consistently aligned parallel to the rolling direction. This controlled orientation was maintained across all materials to mitigate the effects of sheet metal anisotropy. This ensured consistency and minimised variation, thereby isolating the impact of beak geometry on the thickness outcomes. To validate the third assumption, a strain history mapping approach was employed. Specifically, a circle grid analysis was utilised, where 2-mm diameter circles were scribed onto the workpiece with a 1-mm gap between each circle (Fig. 15a).

The scribed surface was placed on the underside of the forming tools, and the changes in the circle diameters were measured at 5-mm intervals throughout the stroke. The deformation of the circles (Fig. 15b) represented the principal strains experienced during forming, which were calculated by considering the change in lengths as shown in Eq. 25.

25 $${\upvarepsilon }_{x}=\text{ln}\left(\frac{{x}_{1}}{{\text{d}}_{0}}\right) \& {\upvarepsilon }_{\text{y}}=\text{ln}\left(\frac{{y}_{1}}{{\text{d}}_{0}}\right)$$

### Results and discussion

A comparative analysis is first presented between the straight and curved beak geometries, followed by an evaluation of part formation across different aspect ratios and material parameters.

**Fig. 16:**
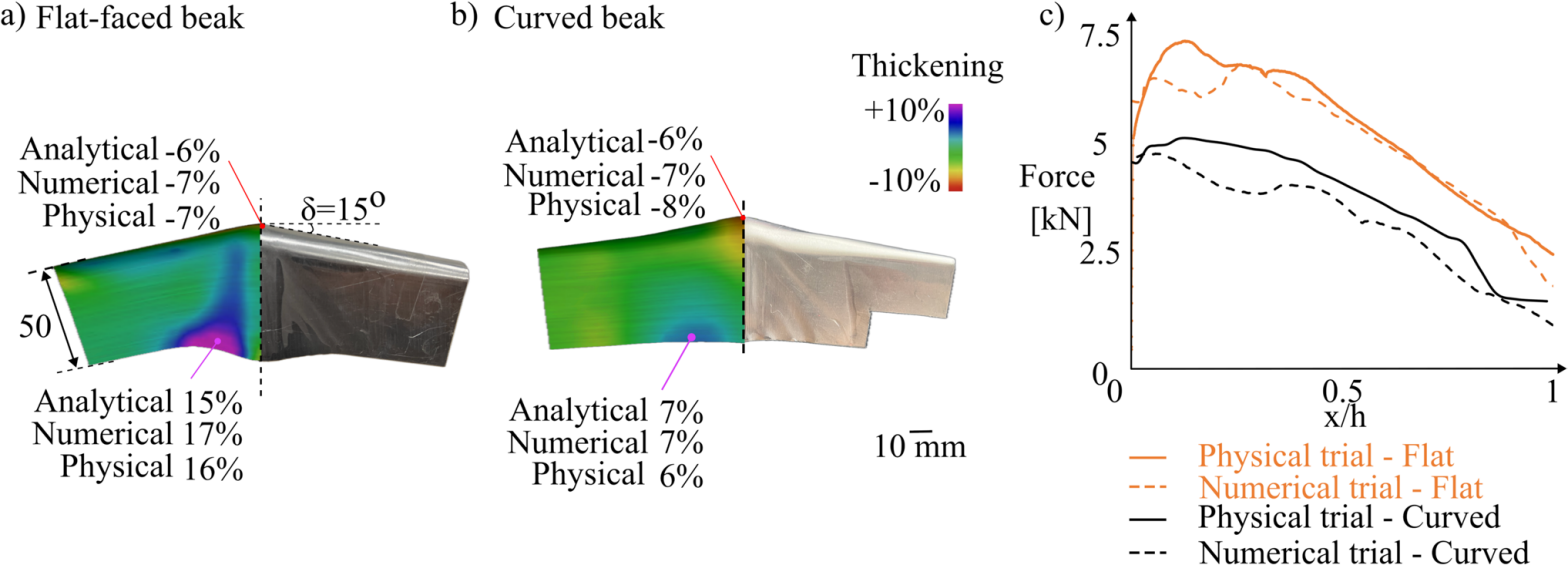
Thickness distribution using (**a**) flat-faced beak [15] and (**b**) curved beak. (**c**) force comparison

Folding–shearing is used to make half a U-channel part using AA1050, a crash angle of 15°, a shear zone angle of 45°, a part height of 50 mm, and a part thickness of 1.5 mm. Figure 16 compares the thickness and force results from the flat-faced beak (Fig. 16a) and the new curved beak (Fig. 16b).

The maximum thinning was observed at the apex while the maximum thickening was observed at the edge of the beak. The thinning remained comparable at 7% versus 8% while using flat-faced or curved beaks; however, the thickening at the sidewall has decreased from 16 to 6%. The numerical model predicts the thinning and thickening to within 12.5% and 16%, respectively, while the analytical model predicts the thinning and thickening to within 25% and 16%, respectively. Figure 16c demonstrates a 27% force reduction while using a curved beak compared to a flat-faced beak geometry. This reduction in force aligns with metal forming principles, where a curvature helps to redistribute the strains across a larger contact area and reduces the localised stress concentration. This, in turn, promotes a more uniform material flow, and in a production setting, it translates directly to lower press tonnage requirements, thus reducing energy consumption. The force curve in Fig. 16c not only shows a lower peak but also a smoother transition, suggesting that curvature-induced strain distribution helps to avoid sudden strain localisation, which can reduce fatigue in tooling. Additionally, a curved beak geometry results in a non-developable folded surface, unlike a flat-faced beak, which remains developable. This inherently introduces in-plane stretching, which deviates from the ideal folding kinematics. The strain effects are more pronounced in regions of high curvature, leading to greater deviation from the ideal folding behaviour. Incorporating this strain contribution in future refinements will enhance the accuracy of the analytical model.

**Fig. 17:**
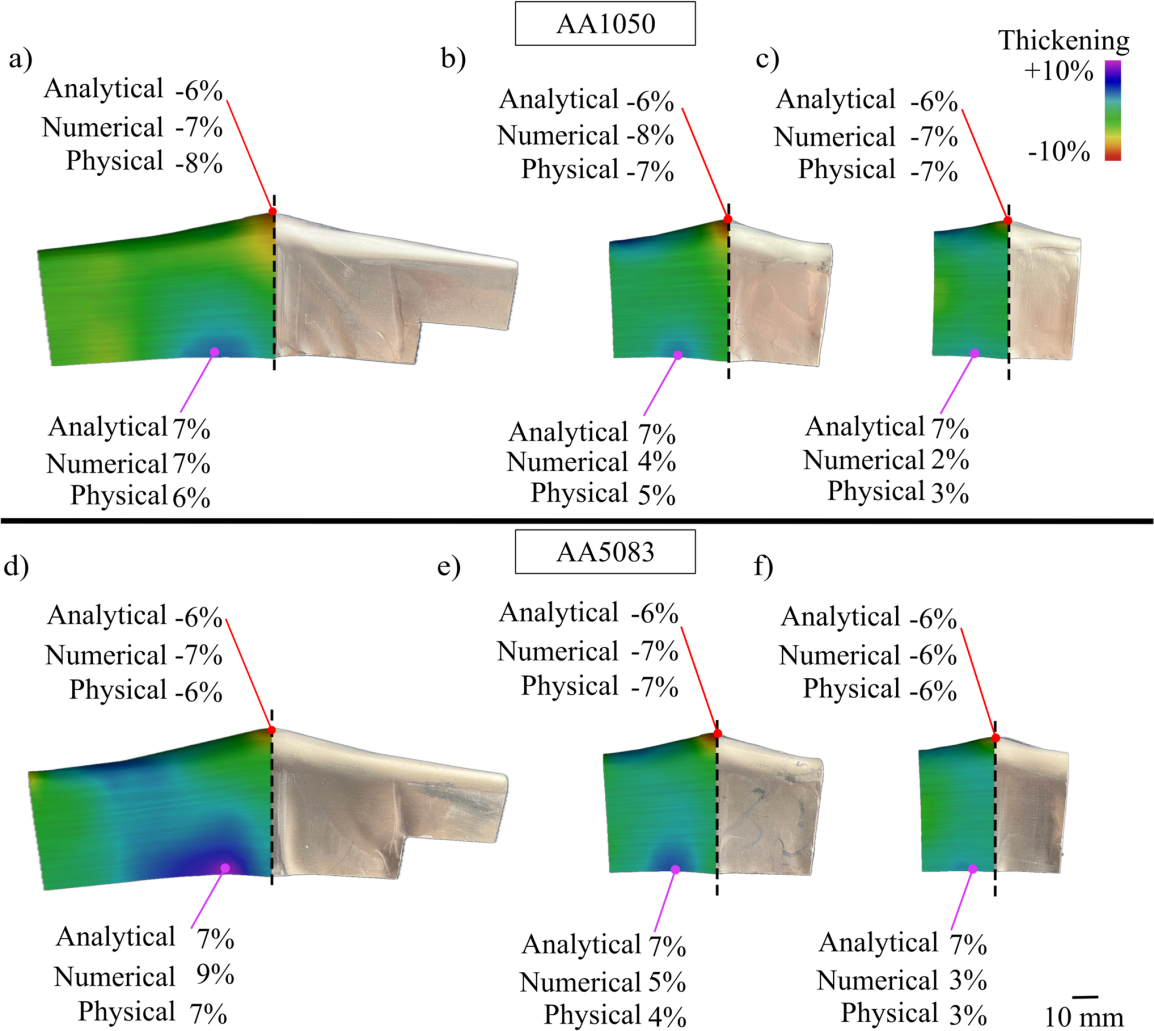
Thickness results using new tools using AA1050 of aspect ratios (**a**) 0.25, (**b**) 0.5, and (**c**) 0.75; and AA5083 of aspect ratios (**d**) 0.25, (**e**) 0.5, and (**f**) 0.75

Figure 17 compares the resulting thickness distribution for both AA1050 and AA5083 across the aspect ratios of 0.25, 0.5, and 0.75 respectively.

The thinning at the apex of the workpiece remains consistent across all six experiments (AA1050: Fig. 17 a, b, and c; AA5083: Fig. 17 d, e, and f) ranging from 6 to 8%. This validates the assumption in the thickness model that the thickness at the apex is independent of the aspect ratio and is only dependent on the curvatures resulting from crash forming and folding. Similarly, the thickening percentage is comparable across materials for the same aspect ratio. For example, thickening of 6% and 7% are observed in AA1050 and AA5083, respectively, for an aspect ratio of 0.25. This reinforces the conclusion that the sidewall thickening is predominantly governed by the curvature induced by the feed radius and the fold line. However, it may also arise from the different strain-hardening behaviours or inherent anisotropy of the materials, which can influence the localised material flow and thereby thickness profiles. Furthermore, as the aspect ratio increases, the volume of material within the beak decreases and, consequently, less thickening is observed. This shows that the analytical model serves as an upper bound, illustrating the worst-case scenario for thickness during the shearing process, and acts as a useful reference for tool design. However, future work will refine the predictions by accounting for material anisotropy and the interplay with aspect ratios.

**Fig. 18:**
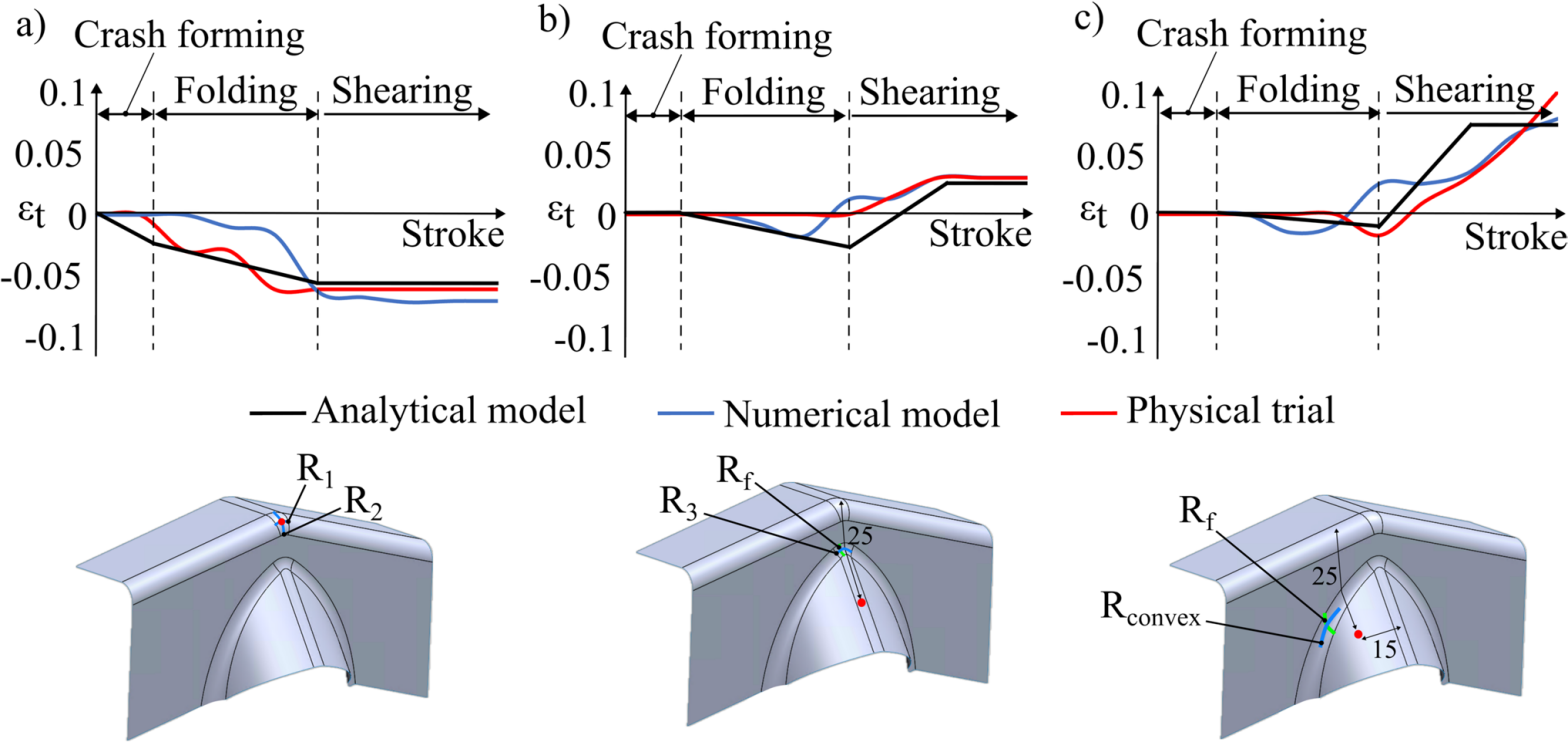
Thickness strain at the (**a**) apex, (**b**) centre of part, and (**c**) side of beak

The final assumption to be validated is that the thickness of the workpiece remains constant after passing through the deformation zone. This is done using a circle grid analysis where the strain history of three points is mapped in Fig. 18. Tracking individual points can offer deeper insights into strain history and potential failure modes. The respective stages are labelled: crash forming, folding, and shearing.

Figure 18a shows that the apex progressively during crash forming and during folding, while the thickness change during shearing is negligible. This trend aligns with the analytical model assumption and confirms that the evolution of thickness is a result of biaxial thinning which is influenced by ${R}_{1}$ and ${R}_{2}$, exclusively. Figure 18b and c track two points on the beak: one at the centre and another at the midpoint of the beak’s sidewall. A similar trend is observed at both these points, where it first experiences tensile strains induced by a convex curvature, governed by ${R}_{3}$ or ${R}_{convex\_max}$, respectively. The strains are subsequently balanced by the compressive strains induced by the concave feed radius, ${R}_{f}$, as the material passes through the deformation zone. Across all three locations, differences between the analytical model, numerical simulations, and physical trials range from about 5% to 25%. The deviation between the analytical model and the physical trial is more pronounced at the bottom of the part due to the anticlastic behaviour of the material [8]. This behaviour is commonly observed in sheet metal forming, where the workpiece experiences higher minor strains at a free edge, thereby leading to thickening. This localised effect is not captured by the analytical model, which assumes a uniform strain distribution through the stroke. However, the model is generally applicable for the majority of the sheet. Evidently, an optimal beak geometry exists for a given shear zone and crash angles. To enable design engineers to rapidly select optimal geometries, the following section presents a design map, illustrating the impact of key process parameters on thickness distribution.

## Optimal beak geometry for shrink corner deformation

For a pure shear deformation, the compressive strains induced by the concave feed radius must be balanced by the tensile strains induced by the convex fold line. However, the ability to achieve a pure shear deformation is limited by the geometric constraints; specifically, the maximum allowable curvature along the fold line is found to be a function of the part geometry, namely, the part height, the shear zone angle, and the crash angle. While this makes achieving a pure shear deformation theoretically unattainable, a near-pure shear condition remains a viable target and is favourable as it allows for an improved thickness distribution. This section presents a design map for optimizing beak geometry by analysing the interplay between feed radius, shear zone angle, and crash angle for a given part height. This map provides a practical guide for engineers to make informed design decisions when targeting a specific shrink corner geometry.

On average, the analytical model of thickness, as validated in the previous section, can predict the final sidewall thickness to within 8.3%, as compared to the physical trials. These margins are considered acceptable for drawing general conclusions from the analytical model. The final thickness across the sidewall is restated in Eq. 25 as:

25 $${\text{t}}_{\text{f}\_\text{sidewall}}={t}_{0}-\frac{{t}_{0}^{2}}{2}\left(\frac{-2}{{R}_{f}}+\frac{1}{{R}_{fold}}\right)$$

where, ${t}_{\text{f}\_\text{sidewall}}$ is the final thickness, ${t}_{0}$ is the initial thickness, ${R}_{f}$ is the feed radius, and ${R}_{fold}$ is the convex radius along the fold line, defined as:

26 $${R}_{fold}=\frac{h(1-sin\theta )}{2{\mathit{cos}}^{2}\theta }$$

where, $h$ is the part height and $\theta$ is the shear zone angle.

**Fig. 19:**
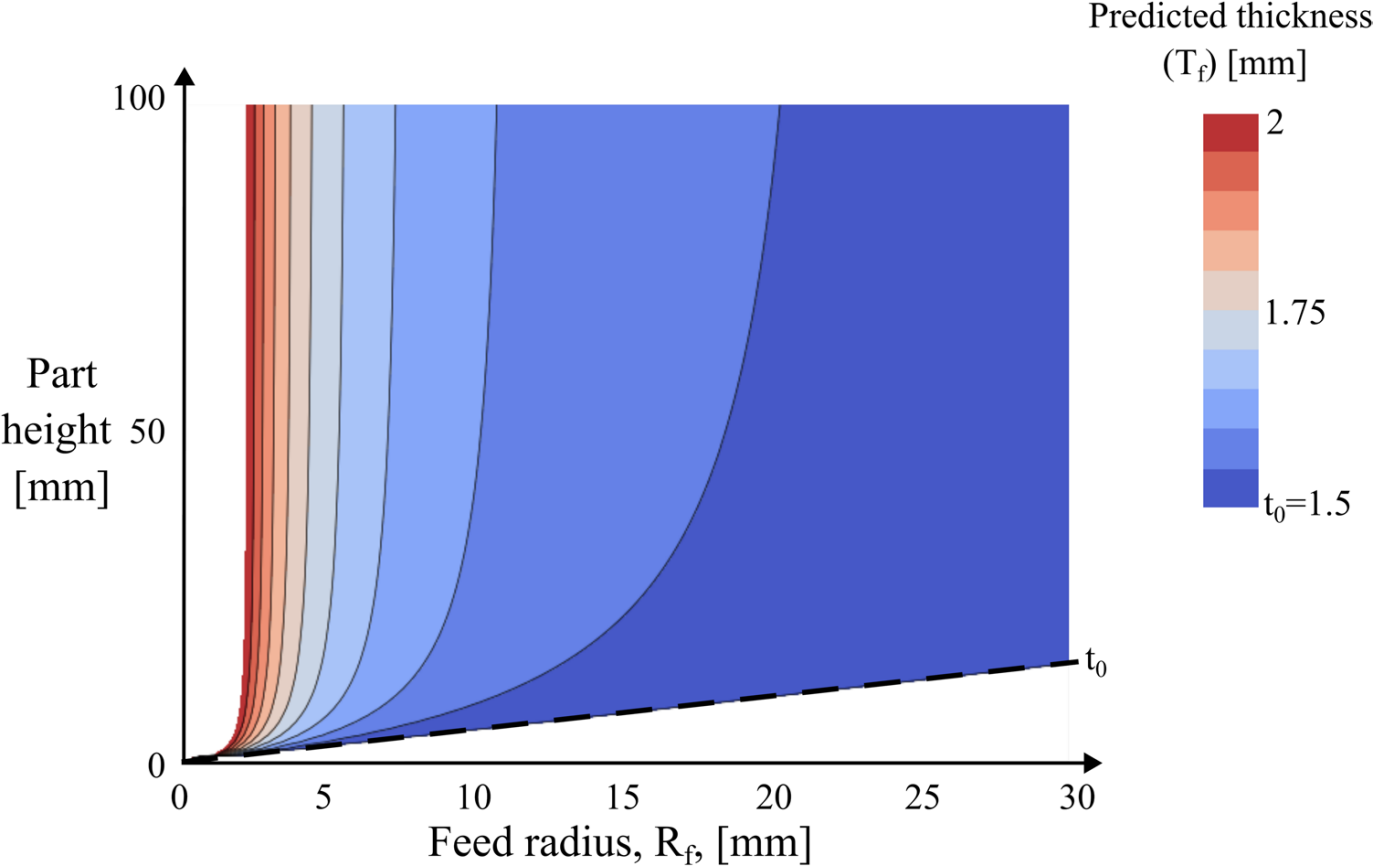
Predicted final thickness across varying feed radii for shear zone angle of $45^\circ$

A key insight from Eq. 25 is that achieving a pure shear deformation requires the feed radius (${R}_{f}$) to be twice the convex radius (${R}_{fold}$). However, ${R}_{fold}$ is dictated by Eq. 26, where the radius is a function of the input parameter of the shear zone angle and the target part height. For a given shear zone angle and part height, the feed radius remains the sole controllable design parameter for a tool design engineer and its impact is demonstrated in Fig. 19.

Figure 19 plots the contours of final thickness (Eq. 25) across a range of feed radius and part heights with a nominal thickness of 1.5 mm and shear zone angle of $45^\circ$. In the contour plot, dark blue areas indicate that the final thickness is closer to nominal thickness, while red areas represent thickening, with the legend extending up to a final thickness of 2 mm, representing a 33% thickness increase. Smaller feed radius results in an increased bending and unbending strain, leading to a more pronounced thickening. Conversely, a larger feed radius decreases the bending and unbending strains, thereby reducing the thickening effect. It is important to note that Fig. 19 uses a shear zone angle of $45^\circ$; however, a change in this value has also been found to directly affect the deformation mechanics [15]. A smaller shear zone angle results in a sharper beak, which in turn increases the material accumulation in the beak, thereby thickening due to increased compressive strains. Conversely, a large shear zone angle produces a broader beak, requiring higher forming forces that induce tensile-dominated thinning, akin to a uniaxial tensile test. This trade-off highlights the balance between geometric precision and material stability and a unique design map is expected for each shear zone angle.

In conclusion, the thickness change along the sidewall is predominantly controlled by the fold line curvature and the feed radius. While the fold line curvature is constrained by geometry, the feed radius can be independently adjusted. A larger feed radius is generally beneficial for balancing the tensile strains induced by the convex fold line. The key takeaway is that optimising this interplay can result in a near pure-shear deformation. Equations 25 and 26 enable engineers to build a design map to define an optimal parameter set instantly, without the need for extensive numerical or physical validation. Balancing these two radii is of crucial importance — an aspect thus far not documented in any folding-shearing literature. To fully understand the potential and limitations of the curved beak design, future investigations will focus on industrial scale forming trials. Such studies are likely to yield valuable insights into real-world process robustness, tooling requirements, and integration with existing manufacturing lines.

## Conclusion

This paper demonstrated the influence of curved beak geometries on thickness distribution in parts made using the folding–shearing process. Half a U-channel part was formed with $15^\circ$ crash angle and a $45^\circ$ shear zone angle. Compared to flat-faced beaks, a curved beak geometry reduced the forming force by 25% and reduced the maximum thickening by 56%. These improvements moved the deformation mode closer to pure shear while enhancing tool longevity.

To summarise, a novel design space was plotted while systematically considering both concave and convex curvatures, resulting in 25 unique beak designs. Each design was evaluated using a numerical model across two aluminium alloys and three aspect ratios. Additionally, an analytical model was developed to predict the thickness as a function of the design parameters. The optimal design featured a negative Gaussian curvature at the deformation zone, which induced tensile and compressive strains along the orthogonal directions. The analytical model predicted thinning at the apex and thickening at the sidewall to within 12.5% and 8.3% for both AA1050 and AA5083, as compared to physical trials. This discrepancy stems from the model’s assumptions that the thickness is only dependent on the geometry of the deformation zone, whereas other factors such as strain hardening, frictional effects, and anisotropic material behaviour will contribute to the deformation mode and are not captured by the analytical model. Despite these limitations, the analytical model serves as a valuable tool for understanding the influence of curvature on the final thickness and offers guidance to tool design engineers to identify the optimal beak design parameters without the need for excessive numerical or physical trials.

Future work will aim to investigate the role of curved beak geometries on a range of complex part geometries, such as stretch corners and cylindrical cups, where failure is often driven by localised thinning or anisotropic-induced earing. For instance, while the beak designs in quadrant 4 induce both compressive and tensile strains, those in quadrant 2 solely induce compressive strains. This symmetry in the design space underscores the potential for developing a broader range of tool configurations tailored to different corner types, hence enhancing the overall versatility of the folding-shearing process. Several avenues for future research remain: a key area of investigation will be the impact of material anisotropy on the forming limits achievable with this process. This will be complemented by expanding the material selection to include ultra-high strength steels, further broadening the scope of the folding–shearing process. Additionally, the feasibility of integrating the folding–shearing process into a multi-step forming route will be examined. This includes combining it with downstream operations such as hemming, flanging, or re-drawing, which could enable the production of complex geometries while leveraging the distinct advantages of each process.
